# The Role of High-Density Lipoprotein in Lowering Risk of Dementia in the Elderly: A Review

**DOI:** 10.7759/cureus.24374

**Published:** 2022-04-22

**Authors:** Anusheel, Supraja N Avula, Ke-li-ta N Joseph, Chibuzor V Onuchukwu, Vishwanath Thondamala, Shashwat Shrivastava, Anusha R Namburi, Lubna Mohammed

**Affiliations:** 1 Public Health, California Institute of Behavioral Neurosciences & Psychology, Fairfield, USA; 2 Vascular Surgery, California Institute of Behavioral Neurosciences & Psychology, Fairfield, USA; 3 Internal Medicine, California Institute of Behavioral Neurosciences & Psychology, Fairfield, USA

**Keywords:** dyslipidemia, apolipoprotein, ageing, hdl, alzheimer's, dementia

## Abstract

Dementia is one of the major causes of disability and hospitalization in the elderly. As far as non-invasive markers of dementia are concerned, we only have age and Apolipoprotein-E (Apo-E) gene, which can be considered as clinically relevant. Modifiable risk factors have been found to be the cause in one-third of the patients who develop dementia. The compatible data supporting the same, in particular for dyslipidemia, is limited, which in turn makes it difficult to devise prevention and interventional methods for both dementia and mild cognitive impairment. Hence, the objective of the review is to summarize the findings on the relation established between the high-density lipoprotein type C( HDL-C) levels and lower the chance of dementia in the elderly, and the possible role of HDL-C as a potential predictive biomarker for cases of dementia in elderly people.

Dyslipidemia, a known risk factor for the occurrence of cardiovascular diseases, seems to be linked to Alzheimer’s disease. Elevated levels of serum cholesterol in mid-adult life increases the risk of dementia in older age. But elevated high-density lipoprotein (HDL) level and its principal apolipoprotein A-I (ApoA-I ) equates with a low risk of dementia in the elderly population

HDL cholesterol has been found to promote endothelial nitric oxide synthase activity which in turn reduces the neural and vascular inflammation and suppresses vascular adhesion thereby exhibiting its vasoprotective function. It has been believed that all these factors have a role to play in the pathogenesis of dementia.

The relation between the higher levels of HDL cholesterol or its key protein component ApoA-I and the lower dementia prevalence in the elderly had been documented in numerous observational studies. Some studies have reported conflicting results. Yet, observational studies measuring the baseline HDL level in middle age found a significant association between HDL level and dementia risk in the elderly, whereas those studies measuring HDL cholesterol level only in old age found no association. Likewise, a significant association between HDL cholesterol and dementia risk has been reported with studies that carry through to 10 years or longer. However, the studies with follow-up of fewer than 10 years had failed to document any such association between HDL cholesterol and dementia.

HDL assays may also be used as a predictive biomarker for dementia patients to target the interventions. Although statins do not target HDL directly but can be an area of interest for dementia.

## Introduction and background

Dementia is a syndrome that causes impairment in memory, thinking, and behavior and affects the ability to perform routine activities. Dementia is one of the leading causes of disability among the elderly population worldwide. The fifth edition of its Diagnostic and Statistical Manual of Mental Disorders (DSM-5) replaces the term “dementia” with major neurocognitive disorder and mild neurocognitive disorder. The impairment must represent a decline from a previously higher level and should be documented both by history and by objective assessment. It is a growing health problem in both developed and developing countries. About 5-8% of the general population aged over 60 years was estimated to be affected by dementia at a given point in time [1]. The exact underlying pathophysiology of dementia remains unclear, even though several risk factors have been identified for dementia. Aging is one of the major risk factors for dementia. Although young-onset cases of dementia were increasingly reported, dementia is a disease of old age. The other major risk factors are apolipoprotein-E (APO-E) genes and a family history of dementia. Many modifiable cardiovascular risk factors were also associated with a higher risk of dementia, like smoking, diabetes, hypertension, midlife obesity, physical inactivity, and high cholesterol [2,3]. Though at present, there is no cure for dementia or drug to alter the progress of the disease, various new treatments are being investigated for dementia, which are in different stages of clinical trials. Dementia has many social implications causing a great amount of stress to the families and caregivers. Also, a high cost is required for the health and long-term care of people with dementia.

Various epidemiological studies in humans and some animal studies support the hypothesis that a high level of high-density lipoprotein (HDL) is associated with lower dementia risk in the elderly. Hence, it can be used as a predictive biomarker for dementia. Low HDL cholesterol, a known risk factor for cardiovascular disease, is also associated with higher dementia risk in the elderly. Cerebrovascular diseases have been suggested to contribute to neuropathological changes in cases of dementia [2]. HDL cholesterol level has many vasoprotective functions and has been positively correlated with cognition in dementia patients [3]. Several mechanisms may lie behind the relation between high cholesterol and dementia [4].

A biomarker is a tool that may indicate the presence or absence of a particular disease, future risk of a disease, or severity of the disease. Many factors are currently in research as a biomarker for dementia risk, like the amount of beta-amyloid in the brain, levels of tau protein in cerebrospinal fluid, and positron emission tomography (PET) imaging of the brain showing glucose metabolism [5]. But from a public health point of view, developing a simple and inexpensive biomarker like a blood test would be of great benefit. Also, a biomarker-based diagnosis of dementia in the future will eventually enable more accurate dementia prevalence estimation.

Study results have been contradicting the possible role of statins in cognition and protection against dementia. Statins, though not directly targeting HDL, may also be of interest for dementia [6]. The purpose of the review is to summarize the findings on the association between HDL cholesterol levels and lower dementia risk in the elderly and the possible role of HDL cholesterol as a potential predictive biomarker for dementia in elderly people.

## Review

Accumulating evidence is available which says that cerebrovascular disease plays a major role in the pathology of dementia patients [2]. Most dementia patients exhibit cerebrovascular dysfunction like microinfarcts, macro infarcts, cerebral amyloid angiopathy (CAA), arteriosclerosis, and atherosclerosis. Reports of larger studies on autopsy like the National Alzheimer’s Coordinating Centre (NACC) and the Religious Orders Study/Memory and Aging Project (ROS/MAP) had reported that the patients with dementia were found to have a greater burden of cerebrovascular disorders [2].

Dyslipidemia, a known risk factor for cardiovascular disease, seems to be connected with Alzheimer’s disease [3]. High serum cholesterol is a well-accepted component of risk for the occurrence of coronary heart disease and stroke. Maximized serum cholesterol in mid-adult life increases the risk of dementia in older age [4]. Many recent observational studies have reported high cholesterol levels as an involved risk factor for dementia also [3,7,8]. But elevated HDL cholesterol level and its principal apolipoprotein A-I (Apo-AI) is associated with low chances of dementia in the elderly.

Research has also shown that the risk or chances of dementia upsurge with the number of vascular risk factors [3]. The significance of cerebral vasculature in dementia is well supported by the connection of cardiovascular disease among the patients with Alzheimer’s disease risk [7]. There are various risk factors like age, gender, physical activity, smoking, elevated blood pressure, dyslipidemia, and diabetes mellitus, which are common among cardiovascular diseases and dementia [8]. Studies also attributed a third of Alzheimer’s disease burden due to modifiable risk factors [8].

Studies have shown that cholesterol intervenes with the degradation process of the amyloid precursor protein and that the apolipoproteins are involved in the metabolism of beta-amyloid [9,10]. Beta-amyloid is an important factor involved in the pathophysiology of dementia. Evidence to support this claim comes from both in vitro and in vivo studies [9,10].

High-density lipoprotein (HDL) plays a major protective role in small vessel disease by taking out the excess cholesterol from the brain. On the other hand, dyslipidemia can also affect dementia through noncerebrovascular mechanisms like interfering with the amyloid degradation process.

HDL cholesterol has many known vasoprotective functions, including advancing endothelial nitric oxide synthase activity, which reduces neuroinflammation, reduces vascular inflammation, and suppresses vascular adhesion. All these factors play a part in the pathogenesis of dementia [11]. The procedure by which HDL cholesterol impacts dementia risk remains unknown.

Four possible distinguishing functions that can safeguard against Alzheimer’s disease have been shown by HDL cholesterol. HDL inhibits the accumulation of cerebral amyloid angiopathy. The cerebrovascular inflammation is induced by amyloid or cytokines repressed by HDL cholesterol. Nitric oxide (NO) production by the endothelial cells is stimulated by HDL cholesterol. The fibrillization of Aβ is inhibited by HDL cholesterol [12].

Out of the many, one key genetic risk component responsible for late-onset Alzheimer's disease is Apolipoprotein-E4 (APOE4), which delays the transport of Aβ from the brain, causing blood-brain barrier breakdown. This, in turn, results in the inflammation of the neural tissues. It is also noteworthy to mention that most of the HDL-like particles in the brain are Apo-E based [13].

**Figure 1:**
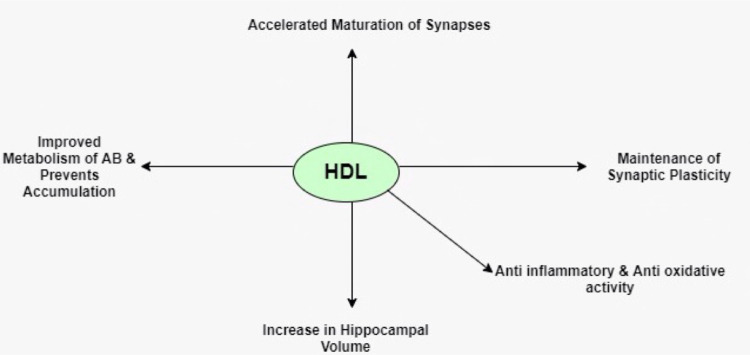
Beneficial effects of HDL in dementia patients AB - Aβ, HDL - high-density lipoprotein

Numerous studies have also been done on animals to explore the relationship between HDL cholesterol and Alzheimer's disease. It has been reported that the ablation of Apo-AI genetically in mice increased the cerebral amyloid activity deposits in the brain, and it worsened the memory in mice, which supported the hypothesis of an association of low levels of HDL cholesterol with dementia [14]. Moreover, HDL-based therapies for Alzheimer's disease mice resulted in improvements in dementia among the mice [15]. Recently, two large genome studies also found a significant association between HDL particle genes with Alzheimer’s disease risk [16,17]. A brief summary of various beneficial effects of HDL in dementia patients has been illustrated in Figure 1

Various epidemiological studies have shown an association between lower dementia risk in the elderly and high HDL levels or Apo-AI, which is a vital protein component of HDL [18-21]. The famous InChianti study also concluded that there were significantly low levels of total cholesterol and HDL cholesterol among patients with dementia. However, on multivariable analysis, a significant association was present only for the lower levels of HDL cholesterol [19]. In a study done on Japanese dementia patients, the level of HDL cholesterol level was significantly lesser in dementia group patients. Apolipoprotein A-I and A-II levels also have decreased in the patients, especially in the vascular dementia group [20]. Cross-sectional studies conducted also revealed that serum HDL levels are found to be remarkably lesser in patients with Alzheimer’s disease [22,23]. In the Copenhagen General Population Study, data from 75,708 revealed that participants with HDL cholesterol levels were notably lowered among the patients with dementia [24]. The study also found out that fewer plasma levels of Apo-E were linked to an elevated risk of future Alzheimer’s disease and all causes of dementia amongst the general population.

In the prospective Honolulu-Aging study, the relation between dementia having serum levels of apolipoprotein A-I (Apo-AI) alone and combined levels with Apolipoprotein E (Apo-E) genotype were examined. Nine hundred and twenty-nine Japanese-American men were followed up for 16 years, and it was found that high plasma Apo-AI at baseline level significantly corresponded with a low risk of dementia in the elderly [25]. Correspondingly, the Baltimore Longitudinal Study of Aging showed high HDL baseline cholesterol protected against cognitive impairment 20 years later [26].

Surprisingly, some observational studies, including the Framingham study and Adult Changes in Thought study, had concluded that there was no significant connection between HDL cholesterol and cognitive impairment [27,28]. In the Framingham study, 1026 subjects from the original group who happened to be alive and didn’t have the stroke and dementia had undergone Apo-E genotyping. The key finding was an incidence of Alzheimer's disease. After adjustment for other risk factors, no significant correlation between the risk for incident Alzheimer's disease and average cholesterol level was found [27]. Another study is known as the ACT Study, which is a prospective population-based group study that also found no statistically significant association between HDL-C and risk of Alzheimer’s disease.

The Three-City Study, a multi-center study conducted in France that enrolled 7053 patients and followed up for seven years, established no relation between HDL-C and dementia. This study reported that there were no considerable connections between the total or low-density lipoprotein (LDL) and dementia or AD in both sexes. However, it was found that less HDL cholesterol and increased triglycerides levels might be involved risk factors of dementia in elderly men [28].

Even so, observational studies measuring the baseline HDL level in middle age found a noteworthy relation between HDL level and risk of dementia [25,29]. Likewise, a significant association between HDL cholesterol and dementia risk has been reported in studies that went on up to 10 years or longer [24,25]. However, the studies with follow-up of below 10 years have failed to document any such association between HDL cholesterol and dementia [27,28,30].

In summary, those studies measuring HDL cholesterol levels only in old age found no association, while longitudinal studies with more than 10 years of follow-up and studies which measured baseline middle age HDL-C levels found a noteworthy association between HDL-C and lower risk of dementia [31,32].

With the maturing of imaging techniques along with improvements in the fluid biomarkers, there has been rapid progress in the identification of biomarkers for dementia in recent years. Huge progress has been made even until the development of HDL-based assays, which could correlate with the cerebrovascular health of the individuals. HDL functions, including inhibiting vascular inflammation, neuroinflammation, Aβ fibrillization, and promoting nitric oxide (NO) production, have the potential to take on as a predictor of Alzheimer’s disease. Populations can be sub-grouped into high-risk populations using predictive biomarkers to target the interventions. Hence, HDL assays may be used as a predictive biomarker for dementia patients to target the interventions. Early measurement of HDL cholesterol, i.e., mid-adult life, may allow for a necessary lifestyle modification of risk factors to prevent dementia. Studies have also shown low mid-adult life HDL cholesterol level as an important predictor of cognitive decline in later stages of life independent from other known and important cardiovascular risk factors such as overweight/obesity, low-density lipoprotein (LDL) cholesterol, triglycerides, hypertension, smoking, and diabetes mellitus, [3].

HDL cholesterol level has been positively correlated with cognition in dementia patients [3]. Additionally, HDL assays can also be used as a prognostic biomarker in dementia patients. Prognostic biomarkers predict the severity of the disease before irreversible damage occurs. It is therefore important to evaluate the role of HDL as both a predictive and prognostic biomarker for dementia patients before irreversible vascular damage occurs. It is also important to evaluate the role of HDL-associated proteins (Apo-AI) as a potential biomarker for dementia patients. Studies have also shown that Apo-AI levels are inversely related to future dementia risk [25,29]. 

Statins are known for their role in minimizing both mortality and morbidity in patients with a high risk of cardiovascular diseases. Statins, though not directly aiming at HDL, might also be of interest for dementia. Few meta-analysis results showed that the use of statins lowers dementia [33,34]. Meta-analysis results showed the use of statins was related to a reduction in the risk of dementia (relevant risk: 0.85). In addition, the dose-response analysis showed the use of statins for the duration of one year was related to dementia risk reduction by 20%, and an incremental increase of statins per 5 mg mean daily dose use was associated with a dementia risk reduction of 11%. Another meta-analyzed data from 11 studies involving over 23,000 participants, who had been consuming statins for between 3-25 years, had reported that those taking statins have a 29% reduction in developing dementia [34]. But randomized controlled trial (RCT) results showed no association [34]. A Cochrane review of statins for the prevention of dementia also showed no benefit [35]. The Cochrane review included double-blind, randomized, placebo-controlled trials in which statins were supervised for a minimum of 12 months for persons who were at risk of dementia in the analysis. The study reported no dissimilarity between placebo groups and statin on five distinct cognitive tests.

## Conclusions

Our paper analyzed free articles, including systematic reviews from several databases. Being a relatively new topic, there should be more research in order to draw parallels between HDL and the course of dementia. Further research should mainly be in the direction of the exact mechanism by which HDL acts as a protective agent in dementia and the cause-effect relationship between the two. A growing body of evidence from epidemiological studies supports the role of HDL cholesterol in lowering the risk of dementia in the elderly. Longitudinal studies with more than 10 years of follow-up and involving a large number of study subjects found a significant association between HDL-C and lower dementia risk. Large genome studies and animal studies also found a significant association between HDL particle genes with dementia disease risk. HDL cholesterol assay can also be used as a predictive biomarker assay for future dementia risk and stratifying the dementia patients for targeted interventions and measuring plasma HDL-C represents a promising simple biomarker for predicting the dementia risk. A predictive biomarker enables the early detection of dementia and gives us a big window of opportunity to alter the progression of the disease by addressing the modifiable risk factors of dementia. Early detection of dementia provides a number of benefits, both for the affected dementia patients and their families. The role of lipid-modifying drugs like statin in the prevention and treatment of dementia needs further evaluation.
